# High risk of misclassification of acute Parvovirus B19 infection into a systemic rheumatic disease

**DOI:** 10.1093/rap/rkae105

**Published:** 2024-09-06

**Authors:** Bernardo D’Onofrio, Giulia Virelli, Elisa Pedrollo, Marta Caprioli, Marta Riva, Daniela Renna, Antonio Tonutti, Nicoletta Luciano, Angela Ceribelli, Elisa Gremese, Maria De Santis, Carlo Selmi

**Affiliations:** Rheumatology and Clinical Immunology, IRCCS Humanitas Research Hospital, Rozzano, Italy; Rheumatology and Clinical Immunology, IRCCS Humanitas Research Hospital, Rozzano, Italy; Rheumatology and Clinical Immunology, IRCCS Humanitas Research Hospital, Rozzano, Italy; Rheumatology and Clinical Immunology, IRCCS Humanitas Research Hospital, Rozzano, Italy; Rheumatology and Clinical Immunology, IRCCS Humanitas Research Hospital, Rozzano, Italy; Rheumatology and Clinical Immunology, IRCCS Humanitas Research Hospital, Rozzano, Italy; Rheumatology and Clinical Immunology, IRCCS Humanitas Research Hospital, Rozzano, Italy; Department of Biomedical Sciences, Humanitas University, Pieve Emanuele, Italy; Rheumatology and Clinical Immunology, IRCCS Humanitas Research Hospital, Rozzano, Italy; Department of Biomedical Sciences, Humanitas University, Pieve Emanuele, Italy; Rheumatology and Clinical Immunology, IRCCS Humanitas Research Hospital, Rozzano, Italy; Department of Biomedical Sciences, Humanitas University, Pieve Emanuele, Italy; Rheumatology and Clinical Immunology, IRCCS Humanitas Research Hospital, Rozzano, Italy; Department of Biomedical Sciences, Humanitas University, Pieve Emanuele, Italy; Rheumatology and Clinical Immunology, IRCCS Humanitas Research Hospital, Rozzano, Italy; Department of Biomedical Sciences, Humanitas University, Pieve Emanuele, Italy; Rheumatology and Clinical Immunology, IRCCS Humanitas Research Hospital, Rozzano, Italy; Department of Biomedical Sciences, Humanitas University, Pieve Emanuele, Italy

**Keywords:** viral arthritis, RA, SLE, systemic autoimmune diseases, classification criteria

## Abstract

**Objectives:**

Parvovirus B19 most frequently causes epidemics of erythema infectiosum in children but also affects adults often leading to rheumatologic manifestations. While the serum profile allows the diagnosis, manifestations may mimic autoimmune conditions. The aim was to evaluate the proportion of patients with acute Parvovirus B19 infection fulfilling classification criteria for rheumatic diseases (RA and SLE).

**Methods:**

We evaluated the clinical and serological features of 54 patients diagnosed with acute Parvovirus B19 infection seeking rheumatological attention between March and June 2024.

**Results:**

The majority of patients were females (78%), with a mean (s.d.) age of 45 (13) years and 54% could not recall any known exposure. Fifty-one/54 (94%) had arthralgia, 27 (50%) arthritis (oligoarthritis in 67% of them), 24 (44%) fever, 19 (35%) skin rash and 7 (13%) purpura. Symptoms resolution generally occurred within 6 weeks. Complement levels were low in 14/33 (42%) tested patients, while the presence of serum ANA, anti-dsDNA, anti-phospholipids and rheumatoid factor was detected in 21/38 (55%), 10/26 (38%), 6/12 (50%) and 5/37 (13%) patients, respectively. Classification criteria for SLE were fulfilled in 93% of ANA-positive patients and RA criteria in 38% of patients with arthritis.

**Conclusions:**

Parvovirus B19 infection manifestations may vary and nearly all patients with positive serum ANA fulfil the classification criteria for SLE. The risk of misclassification in patients with viral infection should not be overlooked.

Key messages

- Almost all ANA-positive patients fulfil the 2019 EULAR/ACR classification criteria for systemic lupus erythematosus.
- More than one-third of patients with arthritis fulfil the 2010 ACR/EULAR classification criteria for rheumatoid arthritis.
- Few patients could experience worse outcomes, especially in the presence of thalassaemia.

## Introduction

Arthralgia, fever, cytopenia and skin rashes are commonly observed during viral infections, particularly SARS-CoV-2, Rubella, Alphaviruses and Parvovirus B19 [1–6]. Since similar manifestations are the hallmarks of systemic autoimmune diseases, such as SLE and RA, the differential diagnosis might be somehow challenging. In that context, classification criteria established by international societies can help within the diagnostic workup [1, 2]. Parvovirus B19 is known for its wide array of clinical presentations [7–11], ranging from erythema infectiosum in school-aged children with variable time trends [10, 12], to severe complications, such as aplastic crisis in individuals with haemolytic disorders and hydrops fetalis in pregnant women [10]. In adults, particularly in females [13], Parvovirus can frequently cause self-limiting musculoskeletal symptoms, ranging from arthralgia, to arthritis, to full-blown autoimmune-like diseases and while a serological diagnosis is sought, the clinical picture may constitute a diagnostic challenge [14–18]. In addition, serum autoantibodies and laboratory abnormalities (e.g. hypocomplementemia) have also been frequently reported during Parvovirus B19 infection [19]. The virus capacity to mimic autoimmune diseases [14–18], may therefore lead to potential misclassification and overtreatments.

We herein describe the clinical and laboratory characteristics and outcomes with specific focus on the possibility to fulfil RA and SLE classification criteria in a large series of patients with acute Parvovirus B19 infection referred to the rheumatology clinic during a recent outbreak in Northern Italy.

## Methods

### Characteristics of the study population

We studied a large series of patients (*n* = 54) consecutively referred to the outpatient clinic of the ImmunoCenter of the Humanitas Research Hospital in Milan for rheumatological complaints between March and June 2024 and diagnosed with acute Parvovirus B19 infection based on serum IgM positivity. Parvovirus B19 serology was requested in those subjects whose signs and symptoms were suspected of viral infection, depending on the rheumatologist’s opinion. Patients were referred to the rheumatologist through the general practitioner or the emergency service of the Humanitas Research Hospital. Demographical, clinical and treatments data were retrospectively collected through medical records, and included symptoms duration, type of musculoskeletal involvement (morning stiffness, number of swollen and tender joint on 44 evaluated—SJC44, TJC44, type of involvement), together with other signs or symptoms suggestive for a systemic autoimmune disease. Serum autoantibodies (ANA, anti-double-strand DNA—dsDNA, anti- ENA, aPL, ANCA, RF, ACPA), complement levels, acute phase reactant and other laboratory tests were also collected, when available.

### Outcomes

The evaluated outcomes included: (i) the fulfilment of the most recent classification criteria for RA and SLE [20, 21]; (ii) the resolution or persistence of symptoms after 6 weeks from disease onset (i.e. disease duration), together with the development of a systemic autoimmune disease; (iii) the development of a more severe clinical picture, defined by the presence of a disease duration longer than 6 weeks and/or the need for hospitalization. The study was conducted according to the declaration of Helsinki and approved by Humanitas Research Hospital Ethical Committee.

### Statistical analysis

Continuous variables were expressed as means and s.d. or median and range. Comparisons between groups were made by means of independent samples t-test or chi square test, as appropriate. Predictors of outcome (i.e. more severe disease) were obtained by logistic regression. All analyses were conducted using SPSS Statistics^®^ Version 29.0, and the significance level was *P* < 0.05.

## Results

### Demographic, clinical and laboratory characteristics of the study population

The demographic and clinical features of the study population are shown in Tables 1 and 2, respectively. Between March and June 2024, a total of 54 patients consecutively evaluated for a suspected autoimmune disease were diagnosed with acute Parvovirus B19 infection. Among them, 51 (92%) had a 2-month follow-up available at the time of data analysis; baseline characteristics of these patients were not significantly different from the entire population (data not shown). Patients were predominantly females (78%), with a mean (s.d.) age of 45 (13) years. Only slightly more than 50% of them reported a known exposure to Parvovirus B19. Twenty-six patients (48%) were referred to the rheumatologist through the emergency department, and 6 of them (11%) required hospitalization.

**Table 1.** Demographic data and comorbidities of the study population

	*n* = 54
Female sex, *n* (%)	42 (79)
Age at inclusion, years, mean (s.d.)	45 (13)
Known virus exposure, *n* (%)	29 (54)
Positive familiar history for ARD, *n* (%)	8 (15)
ER referee, *n* (%)	26 (49)
Hospitalization, *n* (%)	6 (11)
BMI, mean (s.d.)	24 (34)
Hypertension, *n* (%)	7 (13)
Depression, *n* (%)	4 (7)
Fibromyalgia, *n* (%)	3 (6)
Liver disease, *n* (%)	2 (4)
Cancer, *n* (%)	2 (4)
Kidney disease, *n* (%)	1 (2)
Diabetes mellitus, *n* (%)	1 (2)
COPD, *n* (%)	1 (2)
Peptic ulcer, *n* (%)	1 (2)

ARD: autoimmune rheumatic disease; ER: emergency room; COPD: chronic obstructive pulmonary disease.

**Table 2.** Clinical characteristics of the study population

	*n* = 54
Disease duration > 6 weeks, *n* (%)	9 (17)
Arthralgia, *n* (%)	51 (94)
Arthritis, *n* (%)	27 (50)
Mono-	1 (0.4)
Oligo-	18 (67)
Poli-	8 (30)
Wrist arthritis, *n* (%)	16 (30)
Hand arthritis, *n* (%)	18 (33)
Knee arthritis, *n* (%)	3 (6)
Ankle arthritis, *n* (%)	11 (20)
Foot arthritis, *n* (%)	3 (6)
Symmetrical involvement, *n* (%)	38 (70)
MS > 30 min, *n* (%)	29 (54)
SJC44, median (range)	4 (1–14)
TJC44, median (range)	2 (0–34)
PMR-like, *n* (%)	4 (7)
Fever, *n* (%)	24 (44)
Skin rash, *n* (%)	19 (35)
Sicca syndrome, *n* (%)	4 (7)
RP, *n* (%)	3 (6)
Oral/nasal ulcers	3 (6)
Serositis, *n* (%)	2 (4
Alopecia, *n* (%)	1 (2)
NP involvement, *n* (%)	1 (2)

MS: morning stiffness; SJC44: number of swollen joints on 44 evaluated; TJC44: number of tender joints on 44 evaluated; NP: neuropsichiatric.

The most common symptom was inflammatory arthralgia (51/54, 94%), followed by arthritis (27/54, 50%), fever (24/54, 44%), skin rash (19/54, 35%), lymphadenopathies (8/54, 15%) and purpura (7/54, 13%—one of them reporting a ‘gloves and socks’ phenotype). No patients had the classical ‘slapped cheeks’ rash. Notably, despite inflammatory arthritis was documented in a half of the patients at rheumatological evaluation, a higher rate of swollen joints was previously observed by the medical practitioner or the emergency department physician who first evaluated the patients [35/54 (65%) patients]. Among the 27 patients with arthritis, the median (range) number of swollen joints was 4/44 [1–13, 20]. As such, the most common type of articular involvement was oligoarthritis, observed in 18/27 (67%) patients, and a symmetrical distribution was present in 23/27 (85%) patients. Most patients had hands arthritis (18/27, 67%), while wrist, ankle and feet involvement were observed in 16/27 (60%), 11/27 (41%) and 3/27 (11%) patients, respectively. A polymyalgia rheumatica-like distribution was observed in 4/51 (8%) patients with arthralgia; all of them were males (*P* < 0.001) of older age [mean (s.d.) age of 56 (21) years *vs* 43 (10) years in non-polymyalgia patients; *P* = 0.034].

Laboratory tests are displayed in Table 3 and included anaemia in 22/53 (42%) patients, leukopoenia in 10/53 (19%), and thrombocytopenia in 5/53 (9%). No patients developed aplastic anaemia, despite thalassaemia minor was observed in 11/53 (21%) patients. Also, median C-reactive protein (CRP) and erythrocyte sedimentation rate (ESR) levels were 1 (range 0.04–23.2) mg/dl and 21 (range 2–125) mm/h, respectively. Serum proteins analysis showed polyclonal hypergammaglobulinemia in 13/35 (37%) patients. Serum ANA were positive in 21/38 (55%) patients, with a speckled pattern in 9 (43%) and titres higher than 1:160 in 7 (33%). Other tested positive autoantibodies were RF [5/37 (14%)], antiphospholipid antibodies [6/12 (50%)—anti-cardiolipin IgM and lupus anticoagulant in 4/6], and anti-dsDNA [10/26 (39%)]. Of note, half of the patients with anti-dsDNA autoantibodies were negative for ANA while 14/33 (42%) patients had low complement (C3 and/or C4) levels.

**Table 3.** Laboratory and autoantibody profile of the study population

	*n* = 54
Hb < 13 g/dl, *n* (%)	1/53 (41)
WBC < 4000/µl, *n* (%)	10/53 (19)
PMN < 3000/µl, *n* (%)	3/53 (6)
Lymphocytes < 1000/µl, *n* (%)	16/53 (30)
PLT < 300 000/µl, *n* (%)	5/53 (9)
Thalassemic, *n* (%)	11/53 (20.4)
CRP mg/dl, median (range)	0.80 (0.04–23.20)
ESR mm/1h, median (range)	21 (2–125)
Proteinuria, *n* (%)	3/44 (7)
Hypergammaglobulinemia, *n* (%)	13/35 (37)
Hypocomplementemia, *n* (%)	14/33 (26)
C3	13/33 (39)
C4	8/33 (24)
RF positivity, *n* (%)	5/35 (14)
ACPA positivity, *n* (%)	1/35 (3)
ANA positivity, *n* (%)	21/38 (55)
Anti-ENA positivity, *n* (%)	3/35 (9)
Anti-dsDNA positivity, *n* (%)	10/26 (39)
APL positivity, *n* (%)	9/12 (75)
LAC	4/12 (33)
aCL IgM	4/10 (40)
aCL IgG	1/11 (9)
B2GP1 IgM	2/10 (20)
B2GP1 IgG	1/10 (10)

Hb: haemoglobin; WBC: white blood cells; PMN: polymorphonucleates; PLT: platelets; dsDNA: double-strand DNA; aCL: anti-cardiolipin; B2GP1: anti-B2glycoprotein1.

All but nine patients (83%) had symptom resolution within 6 weeks from symptoms onset. Recovery occurred spontaneously in eight (15%) patients, whereas the others were treated with glucocorticoids and/or non-steroideal anti-inflammatory drugs. After 8 weeks from symptoms appearance, only one patient had persisting symptoms (arthralgia) and was diagnosed with Sjögren syndrome based on the presence of pre-existent sicca symptoms with positive Schirmer test and serum anti-Ro/SSA antibodies.

## Outcomes

**Figure 1.**
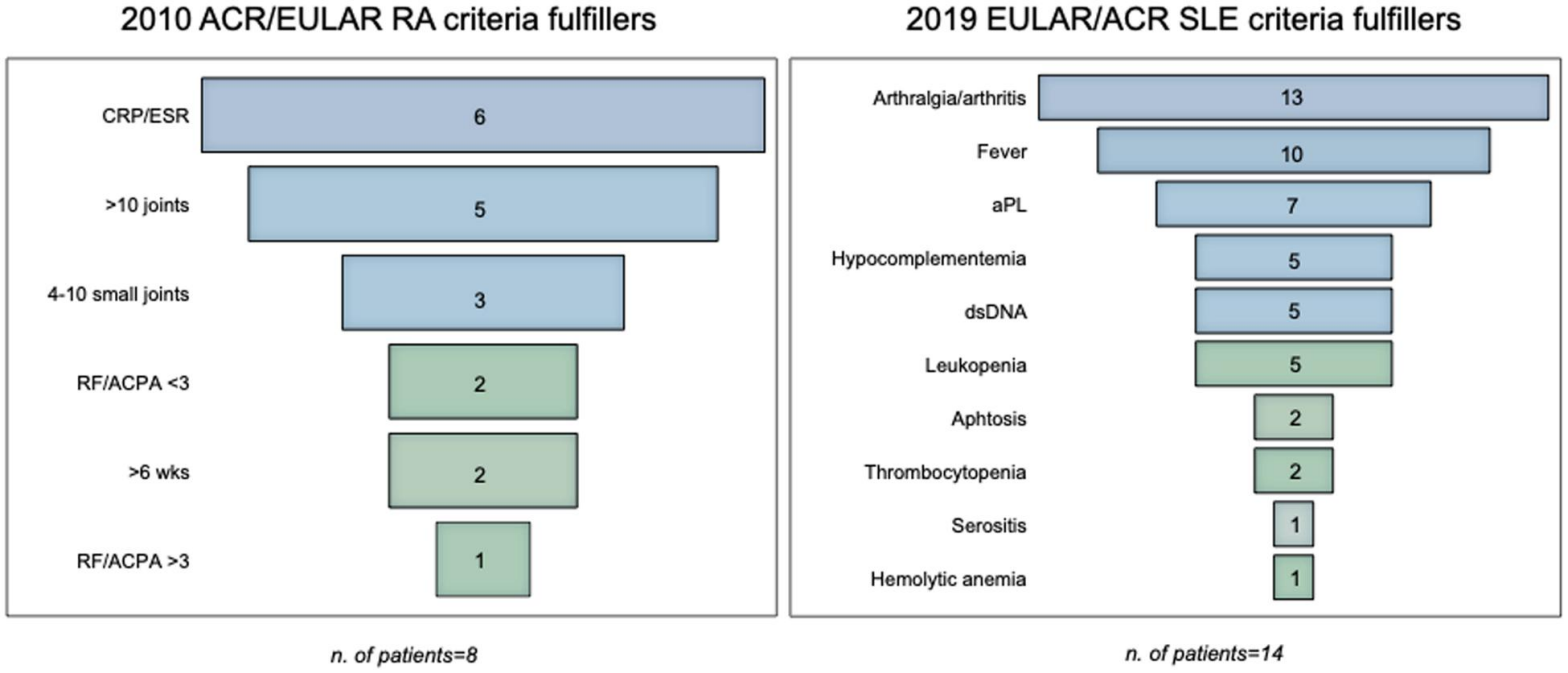
Clinical and laboratory characteristics of patients fulfilling RA and SLE classification criteria. dsDNA: anti-dsDNA antibodies

At the baseline, we applied classification criteria for autoimmune systemic diseases [20, 21] to all patients (Fig. 1). Among cases who fulfilled the entry criterion (i.e. serum ANA positivity) and were tested for other autoantibodies and complement (*n* = 15), the 2019 EULAR/ACR criteria for SLE were met in 14 (93%) patients. With regard to RA, 8 of the 21 patients (38%) with at least 1 swollen joint (entry criterion for 2010 ACR/EULAR criteria for RA) and tested for autoantibodies fulfilled the classification criteria, 5 of which with negative RF and ACPA.

In the overall cohort, 14 (26%) patients were hospitalized and/or had a disease duration of >6 weeks. Through logistic regression, the only predictors of a more severe disease were the presence of lymphadenopathies [β = 1.925 (1.375–34.144); *P* = 0.019] and thalassaemia [β = 1.629 (1.239–20.990); *P* = 0.027]. A similar trend was also observed for purpura [β = 1.596 (0.946–25.737); *P* = 0.058], leukopoenia [β = 1.329 (0.894–15.960); *P* = 0.071] and thrombocytopenia [β = 1.618 (0.746–34.127); *P* = 0.097]. We separately evaluated the demographic and clinical characteristics of patients with acute Parvovirus infection requiring hospitalization (Supplementary Table S1, available at *Rheumatology Advances in Practice* online). Despite the small numbers, we observed that comorbid patients were more often hospitalized (for peptic ulcer, kidney disease and diabetes mellitus: 1 (17%) hospitalized patients *vs* no non-hospitalized patients). Also, the presence of specific clinical and laboratory features were associated with hospitalization [purpura: 3/7 (43%) hospitalized *vs* 3/47 (6%) non-hospitalized, *P* = 0.004; ankle arthritis: 3/11 (27%) hospitalized *vs* 0 non-hospitalized, *P* = 0.027; low white blood cells count: 4/10 (40%) hospitalized *vs* 2/43 (5%) non-hospitalized, *P* = 0.001; low polymorphonucleates count: 2/3 (66.7%) *vs* 4/50 (8%), *P* = 0.002; lymphocytes count: 5/15 (21%) *vs* 1/37 (3%), *P* = 0.003; low platelet count: 3/5 (60%) *vs* 3/48 (6%), *P* < 0.001]. Other laboratory abnormalities were more commonly observed in hospitalized patients (low C4 levels, anti-cardiolipin IgM and anti-B2glycoprotein1 IgG positivity) but data should be interpretated with caution, since patients admitted to the ward were more frequently tested for autoantibodies, as well as those with a higher suspicion of autoimmune disease according to rheumatologist’s opinion (*data not shown*). With regard to treatment, no differences were found between hospitalized and non-hospitalized patients. However, in prednisone users, the mean (s.d.) doses were significantly higher in hospitalized subjects [100 (129) mg/day in hospitalized *vs* 19 (11) mg/day in non-hospitalized; *P* = 0.005].

By the consideration that patients with erythrocyte function impairment might develop a more profound anaemia during Parvovirus infection [10], and the higher hospitalization rate or symptom duration in the case of thalassaemia, we compared thalassemic and non-thalassemic patients (Table 4). Polyarthritis and purpura were significantly more frequent in thalassemic-trait carriers compared with the others [for polyarthritis: 5/7 (71%) patients with thalassemia *vs* 3/19 (16%) patients without thalassaemia; *P* = 0.006; for purpura: 4/11 (36%) patients with thalassaemia *vs* 3/42 (7%) patients without thalassaemia; *P* = 0.011]. The two subgroups of patients displayed differences in laboratory profile also. Particularly, thalassemic patients had more often low complement levels (i.e. C3) and anti-B2glycoprotein1 IgM and IgG antibody positivity [low complement levels in 4/5 (80%) thalassemic *vs* 10/28 (36%) non-thalassemic subjects; *P* = 0.065; low C3 levels in 4/5 (80%) thalassemic *vs* 9/28 (32%) non-thalassemic subjects; *P* = 0.044; anti-B2glycoprotein1 IgM positivity in 1/1 thalassemic *vs* 1/9 (11%) non-thalassemic subjects; *P*= 0.035; anti-B2glycoprotein1 IgG positivity in 1/1 thalassemic *vs* 0/9 non-thalassemic subjects; *P* = 0.002]. Patients with thalassaemia also tended to be more frequently treated with glucocorticoids [7/11 (64%) in thalassaemia *vs* 15/42 (36%) in non-thalassaemia; *P* = 0.094].

**Table 4.** Characteristics of the study population according to the presence of thalassemic trait

	Thalassemic	Non-thalassemic	*P*
	*n* = 11	*n* = 42	
Female sex, *n* (%)	8 (73)	34 (81)	0.549
Age at inclusion, years, mean (s.d.)	47.7 (15)	44.4 (12)	0.442
Smokers, *n* (%)	0 (0)	2 (5)	0.461
Disease duration > 6 weeks, *n* (%)	4 (36)	5 (12)	0.054
Arthralgia, *n* (%)	11 (100)	39 (93)	0.361
Arthritis, *n* (%)	7 (64)	19 (45)	0.277
Mono-	0/7 (0)	1/19 (5)	0.536
Oligo-	2/7 (29)	15/19 (79)	**0.017**
Poli-	5/7 (71)	3/19 (16)	**0.006**
Fever, *n* (%)	6 (55)	18 (43)	0.488
Skin rash, *n* (%)	3 (27)	16 (38)	0.505
Purpura, *n* (%)	4 (36)	3 (7)	**0.011**
WBC < 4000/µl, *n* (%)	2 (18)	8 (19)	0.948
PMN < 3000/µl, *n* (%)	0 (0)	3 (7)	0.361
Lymphocytes < 1000/µl, *n* (%)	3 (27)	13 (31)	0.813
PLT < 300 000/µl, *n* (%)	2 (18)	3 (7)	0.265
Hypocomplementemia, *n* (%)	4/5 (80)	10/28 (36)	0.065
C3	4/5 (80)	9/28 (32)	**0.044**
C4	2/5 (40)	6/28 (21)	0.372
RF positivity, *n* (%)	2/6 (33)	3/31 (10)	0.121
ANA positivity, *n* (%)	2/5 (40)	19/33 (58)	0.461
Anti-dsDNA positivity, *n* (%)	2/4 (50)	8/22 (36)	0.606
APL positivity, *n* (%)	1/1 (100)	8/11 (73)	0.546
PDN use, *n* (%)	7 (64)	15 (36)	0.094

Bold text indicates *P* < 0.05.

WBC: white blood cells; PMN: polymorphonucleates; PLT: platelets; dsDNA: double-strand DNA; PDN: prednisone.

## Discussion

We report that the majority of adult patients with rheumatological manifestations during acute Parvovirus B19 infection may fulfil the classification criteria for SLE and RA and as only half of the patients were aware of the virus exposure within the family or the work environment the risk of misdiagnosis and overtreatment is relevant. When the clinical outcomes were evaluated, we observed that the presence of thalassaemia minor together with specific clinical manifestations at the onset were associated with a prolonged disease duration or hospitalization.

Our study took advantage of a recent surge of cases in the Northern Italian regions but no data are available with regard to infection oscillation in Italy; however, recent reports described an increased prevalence of acute Parvovirus infection across Europe [22–24]. Epidemiological studies report that Parvovirus outbreaks occur according to a seasonal pattern, with most cases presenting in spring with larger epidemics occurring every 1–4 years [25]. Also, preventive measures and restrictions due to SARS-CoV-2 pandemic had a profound impact on the increase of airway viruses transmission across many countries, including Northern Italy [26–28] in the past 4 years and may thus explain the recent number of incident cases.

The main finding of the study is that there is a solid risk of misclassification of patients into a systemic autoimmune rheumatic disease, such as RA and SLE, in the presence of Parvovirus B19 infection. Indeed, when the necessary criterion [20, 21], was met (i.e. serum ANA and at least 1 swollen join detected for SLE and RA, respectively), more than 90% and almost 40% of patients with acute infection could be classified with SLE and RA. Of interest, the largest number of patients classified with RA had seronegative disease and, intriguingly, previous works have speculated that the major chance to achieve drug-free remission for patients with autoantibody-negative arthritis might depend to an initial misclassification of viral arthritis into RA [29]. Of note, 2010 ACR/EULAR criteria suggest classifying patients with RA in the presence of at least one involved joint but only after the exclusion of other causes of arthritis [20]. Thus, when very early arthritis occurs, especially if lacking for autoantibodies, viral arthritis should always been ruled out, as suggested in other studies too [6, 30].

Parvovirus can cause musculoskeletal symptoms, ranging from arthralgia to inflammatory arthritis, and previous reports described RA-like polyarthritis as the most common type of joint manifestation, especially involving the hands and wrists [9, 17, 18]. In our study, although hands were the most frequently involved joints, we observed a higher prevalence of oligoarticular disease instead. Although cases of chronic or recurrent forms have been rarely described, Parvovirus-induced arthritis generally displays a fast, self-limiting, course [18], and the referral to the rheumatologist might take longer than in primary care, even in the presence of an overt clinical picture [31]. We observed a 30% discrepancy between the occurrence of arthritis detected by the non-rheumatologist and the rheumatologist, with the latter following the former by a maximum of one day and this may suggest rapid changes in the clinical manifestations of the joint involvement (i.e. a rapid exhaustion of joint swelling).

Parvovirus B19 infection in adults is associated with other clinical manifestations, including rash, purpura and fever, together with abnormal laboratory findings [1, 2, 9, 15, 16, 18]. Serum autoantibodies (ANA, anti-dsDNA, aPL) and low complement levels are strongly associated with SLE [4–6], and this similarity is mirrored by the immune complexes proposed as one of the pathogenic mechanisms linked to Parvovirus-induced joint symptoms. While RA generally affects women in their 50s, SLE often occurs in women of childbearing age. In the present study, Parvovirus infection is mostly documented in women with a mean age of 45 years, so that the differential diagnosis with RA or SLE might be epidemiologically challenging.

Clinical consequences of infection might be severe therefore requiring aggressive therapies [14, 32–34]. In the present study, none of our patients required a disease-modifying therapy, and only a minority of them were hospitalized. We have reported for the first time that the main factors linked to hospitalization were the presence of comorbidities, such as diabetes mellitus and liver or renal diseases, and the occurrence of specific clinical manifestations, particularly purpura, ankle arthritis and mono-arthritis, low white-blood, red-blood cells and platelet count and lymphadenopathies.

Despite not observing a worrying disease course in most of our patients, beta-thalassemic trait carriers were reported to have a more severe clinical picture, characterized by polyarticular disease, purpura and a higher glucocorticoids use. Parvovirus B19 infects erythrocyte progenitor cells, causing an arrest in erythropoiesis. This may lead to severe anaemia in susceptible subjects such as immunocompromised hosts or individuals with a short half-life of red blood cells (e.g. thalassemic subjects) [25]. Moreover, the seroprevalence and Parvovirus B19 DNA have been detected to be higher in thalassemic patients [35]. On the other side, the prevalence of beta-thalassaemia was found higher in our cohort (20%) compared with that one observed in the general population of Northern Italy (7–10%) [36]. This might suggest that the presence of red-blood cells abnormalities could predispose not only to a worse disease course but also to a greater risk of infection, and this is observed also for patients with rheumatological manifestations.

While we believe that our study reports some observations of clinical relevance, we are aware of the limitations, such as the fact that not all patients were tested for autoantibodies and complement levels, thus reducing the opportunity to classify them into a systemic rheumatic disease. Also, no patients have a laboratory profile at the 2-month follow-up visit, nor a precise clinimetric, and almost none has a longer follow-up. However, none except one was diagnosed with a rheumatological disease after infection, thus making a longer follow-up unnecessary.

In conclusion, we believe that the risk of RA- or lupus-like syndrome should not be overlooked in patients with acute Parvovirus B19 infection as a large proportion of them could fulfil criteria leading to misclassification. Rheumatologists should therefore be aware of the possible mimicking role of Parvovirus B19, in order to avoid diagnostic pitfalls and overtreatments. The impact of comorbidities and thalassaemia in determining the severity of the infection should be evaluated in larger cohorts.

## Supplementary Material

rkae105_Supplementary_Data

## Data Availability

The datasets used and/or analysed during the current study are available from the corresponding author upon appropriate request.
