# Supplementary material for: High risk of misclassification of acute Parvovirus B19 infection into a systemic rheumatic disease
Source: Rheumatol Adv Pract. 2024 Sep 6;8(3):rkae105. doi: 10.1093/rap/rkae105 (PMC11387767; doi:10.1093/rap/rkae105)
Supplement: rkae105_Supplementary_Data [file rkae105_supplementary_data.pdf]

Supplementary Table 1. Comparison between hospitalized and non-hospitalilzed patients.

	Hospitalized n=6	Non-hospitalized n=48	p
Female sex, n. (%)	4 (67)	38 (79)	0.487
Age at inclusion, years, mean (SD)	49.7 (16)	44.3 (12)	0.338
Smokers, n. (%)	0 (0)	2 (4)	0.610
Hypertension, n. (%)	2 (33)	5 (10)	0.115
Depression, n. (%)	1 (17)	3 (6)	0.358
Peptic ulcer, n. (%)	1 (17)	0 (0)	**0.004**
Liver disease, n. (%)	1 (17)	1 (2)	0.075
Kidney disease, n. (%)	1 (17)	0 (0)	**0.004**
Diabetes mellitus, n. (%)	1 (17)	0 (0)	**0.004**
Cancer, n. (%)	1 (17)	1 (2)	0.075
Arthralgia, n. (%)	4 (67)	47 (98)	**0.002**
Arthritis, n. (%)<br>- Monoarthritis, n. (%)<br>- Ankle arthritis, n. (%)	3 (50)<br>- 1/3 (33)<br>- 3/3 (100)	24 (50)<br>- 0 (0)<br>- 8/24 (33)	1.000<br>**0.004**<br>**0.027**
Fever, n. (%)	2 (33)	22 (49)	0.561
Skin rash, n. (%)	3 (27%)	16 (38)	0.505
Purpura, n. (%)	3 (50)	4 (8)	**0.004**
Lymphadenomegalia, n. (%)	3 (50)	5 (10)	**0.010**
Hb<13 g/dL, n. (%)<br>- Hb levels, g/dL, mean (SD)	4 (67) -<br>9.7 (3)	18/47 (38)<br>- 12.3 (2)	0.184<br>**<0.001**
WBC<4000/uL, n. (%)	5 (67)	6/47 (13)	**0.001**
PMN<3000/uL, n. (%)	2 (33)	1/47 (2)	**0.002**
Lymphocytes<1000/uL, n. (%)	5 (83)	11 (23)	**0.003**
PLT<300000/uL, n. (%)	3 (50%)	2 (4)	**<0.001**
Hypocomplementemia, n. (%)<br>- C3, n. (%)<br>- C4, n. (%)	4 (67)<br>- 4 (67)<br>- 4 (67)	10/27 (37)<br>- 9/27 (33)<br>- 4/28 (15)	0.184<br>0.131<br>**0.007**
RF positivity, n. (%)	2 (33)	3/31 (10)	0.121
ANA positivity, n. (%)	5 (83)	16/32 (50)	0.132

Anti-dsDNA positivity, n. (%)	2 (33)	8/20 (40)	0.768
APL positivity, n. (%)	2/2 (100)	7/10 (70)	0.371
PDN use, n. (%)	3 (20)	20 (41)	0.697
Hb=haemoglobin; WBC=white blood cells; PMN=polymorphonucleates; PLT=platelets; RF=rheumatoid factor; ANA=anti-nuclear antibodies; dsDNA=double-strand DNA; APL=antiphospholipids; PDN=prednisone.			
